# Technical Feasibility of the Endoscopic Endonasal Approach for Clipping an Ophthalmic Artery Aneurysm With an Origin Lateral to the Optic Nerve: A Case Report

**DOI:** 10.7759/cureus.109605

**Published:** 2026-05-25

**Authors:** Lydia Kaoutzani, Mohamed Ben Omran, Dan-Victor Giurgiutiu, James K Byrd, Fernando Vale, M. Salman Ali

**Affiliations:** 1 Neurosurgery, Wellstar Medical College of Georgia Health, Augusta, USA; 2 Anesthesiology and Perioperative Medicine, Wellstar Medical College of Georgia Health, Augusta, USA; 3 Neurology, Wellstar Medical College of Georgia Health, Augusta, USA; 4 Otolaryngology - Head and Neck Surgery, Wellstar Medical College of Georgia Health, Augusta, USA

**Keywords:** cerebral aneurysm, cerebral aneurysm surgery, clip ligation, endoscopic endonasal approach, ophthalmic aneurysm

## Abstract

Ophthalmic artery aneurysms (OAAs) present a challenge for microsurgical clip ligation given their close proximity to critical structures. Endovascular management of OAAs is usually the preferred treatment option; however, the risk of recurrence remains a concern. OAAs have previously been clipped via an endoscopic endonasal approach (EEA). To our knowledge, this is the first report exploring the technical feasibility of EEA for clipping an OAA with an origin lateral to the optic nerve and reviewing the literature on all cerebral aneurysms (CAs) clipped via an EEA.

This is a case report of a 36-year-old female with an unruptured left OAA measuring 7 × 7 mm with a 4 mm neck lateral to the optic nerve. She also had a right 2 × 3 mm OAA. Given her young age and bilateral OAAs, we opted for clip ligation of the left OAA to avoid placement of bilateral flow-diverter stents. After extensive discussion with the patient, she underwent successful clip ligation of the left OAA via an expanded EEA approach. In well-selected cases, EEA provides a safe and direct corridor for clipping OAAs with an origin lateral to the optic nerve.

## Introduction

Ophthalmic artery aneurysms (OAAs) are relatively rare among intracranial aneurysms. Their close relationship with the ophthalmic artery, optic and oculomotor nerves, and anterior clinoid process makes them challenging to surgically clip. Proximal control can also be difficult because of the close proximity of the distal dural ring. The space between the distal dural ring and the anterior clinoid process is small, and the placement of a surgical clip is extremely challenging. Endovascular techniques provide a safer treatment option but carry risks of recurrence and commit younger patients to antiplatelet therapy [1-5].

The endoscopic endonasal approach (EEA) provides an excellent but complex corridor for clipping these cerebral aneurysms (CAs). The EEA first encounters the medial side of the optic nerve. Therefore, a lateral aneurysm is located on the far side of the nerve from an EEA, which makes treatment more challenging. Previous case reports have shown the efficacy of EEA for medially projecting OAAs [6-9]. To our knowledge, this is the first report exploring the technical feasibility of EEA for clipping an OAA with an origin lateral to the optic nerve, as well as reviewing the literature on all CAs clipped via an EEA.

## Case presentation

Methods

This is a case report of a 36-year-old female with bilateral OAAs. The aneurysms were discovered incidentally following a motor vehicle accident. She had a large left 7 × 7 mm OAA with a 4 mm neck and an origin lateral to the optic nerve, as well as a smaller right 2 × 3 mm OAA. Figure 1 demonstrates an axial computed tomography (CT) digital reconstruction showing a large OAA with a daughter sac. Figure 2 shows a lateral cerebral angiogram demonstrating the relationship between the aneurysm and the ophthalmic artery. Due to her young age and bilateral CAs, she was not considered an ideal candidate for a flow-diverter stent. After discussion with the patient, it was decided to proceed with surgical clipping. Although an open transcranial approach was feasible, the patient opted for an EEA approach. 

**Figure 1:**
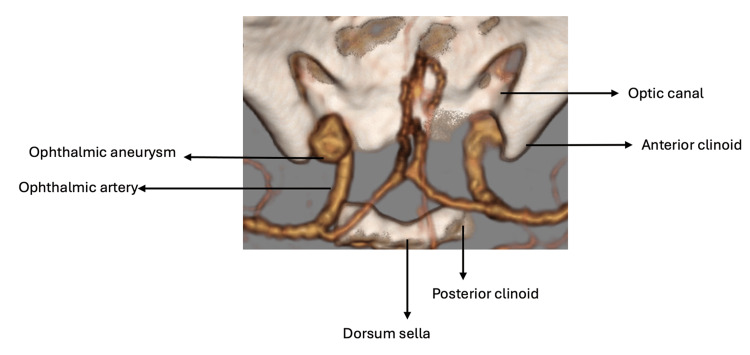
Axial computed tomography digital reconstruction demonstrating a large ophthalmic artery aneurysm with a daughter sac.

**Figure 2:**
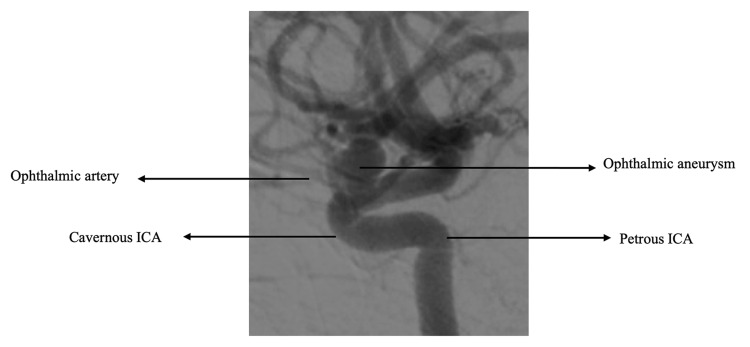
Lateral digital subtraction angiography of the right internal carotid artery injection.

Literature review

Prior studies have demonstrated the successful clipping of 35 CAs of both the anterior and posterior circulation via an EEA (Table 1). These surgeries were performed at a total of nine institutions, resulting in 12 publications. CAs involving the following anterior circulation arteries - superior hypophyseal, anterior communicating, paraclinoid, and ophthalmic - have been clipped via an EEA [6-13]. Posterior circulation CAs, including those involving the basilar artery bifurcation, posterior cerebral artery, posterior inferior cerebellar artery, and vertebral artery, have also been successfully clipped via an EEA [7,14-17].

**Table 1:** Literature review of prior clipping of cerebral aneurysms in both the anterior and posterior circulation using an endoscopic endonasal approach.

Author	Institution	Aneurysm Location/Size (mm)	Growth Direction
Gardner et al. (2015) [7]	University of Pittsburgh Medical Center	Ophthalmic/3.5	Inferior Medial
		Posterior cerebral artery/19	Ventral brainstem surface
		Superior hypophyseal/5	Medial
		Basilar apex/9	Posterosuperior
		Ophthalmic/giant and ophthalmic/5	Superomedial
		Ophthalmic/6	Medial
		Ophthalmic/7	Superomedial
		Ophthalmic/4	Medial
		Superior Hypophyseal/11	Medial
		Superior Hypophyseal/n.a	Inferomedial/n.a.
Kassam et al. (2007) [10]	University of Pittsburgh Medical Center	Superior hypophyseal/5	Medial
Kitano and Taneda (2007) [11]	Kinki University School of Medicine	Anterior communicating/n.a	n.a.
Froelich et al. (2011) [12]	Strasbourg University Hospital	Anterior communicating/7	Superior
Germanwala and Zanation (2011) [6]	University of North Carolina School of Medicine	Ophthalmic/5	Superomedial
		Paraclinoid/10	Posteromedial
Xiao et al. (2018) [8]	The First Affiliated Hospital of Nanchang University	Anterior communicating/7.2	Superomedial
		Paraclinoid/7.2, anterior communicating/3.1	Superolateral, inferomedial
		Paraclinoid/10	Anteromedial
		Anterior communicating/3.5	Anteromedial
		Anterior communicating/5.7	Superomedial
		Anterior communicating/2.8	Superomedial
		Paraclinoid aneurysm/4.2, ophthalmic aneurysm/2.2	Superior, superomedial
Bao et al. (2023) [9]	The First Affiliated Hospital of Nanchang University	Ophthalmic aneurysm/2.2	Superomedial
		Paraclinoid aneurysm/4.2	Lateral
Kassam et al. (2006) [15]	University of Pittsburgh Medical Center	Vertebral artery/11	Ventral brainstem surface
Enseñat et al. (2011) [14]	Faculty of Medicine, University of Barcelona	Posterior inferior cerebellar artery/1.2	Ventral brainstem surface
Drazin et al. (2012) [17]	Loyola University School of Medicine, University of North Carolina School of Medicine	Basilar trunk/4	Ventral brainstem surface
Somanna et al. (2015) [16]	National Institute of Mental Health and Neurosciences, India	Basilar apex/7	Superior and posterior
		Basilar apex/4	Superior
		Basilar trunk/4.94	Left
		Posterior cerebral/7	Superior

Results

Anesthesia

The patient was premedicated with a 2 mg intravenous bolus of midazolam in the holding area before being transferred to the operating room. Two large-bore peripheral intravenous lines and a left radial arterial line were inserted prior to the induction of anesthesia. Intraoperative monitoring included a five-lead electrocardiogram, pulse oximetry, invasive arterial blood pressure, temperature, and capnography. General anesthesia was induced slowly with intravenous fentanyl citrate 150 μg, propofol 2.5 mg/kg, lidocaine 1 mg/kg, and succinylcholine 1.5 mg/kg. The patient was intubated with a size 7 endotracheal tube using video laryngoscopy, and hemodynamics remained stable throughout induction. Anesthesia maintenance was achieved with sevoflurane at 0.5 minimum alveolar concentration, propofol (100-150 μg/kg/min), and remifentanil (0.1-0.2 μg/kg/min) administered as titrated infusions.

Throughout the procedure, hemodynamic stability and a mean arterial pressure of 70-85 mmHg were maintained. At the end of the procedure, the patient was extubated and transferred to the neurosurgery intensive care unit for postoperative monitoring. She remained neurologically intact and was discharged from the hospital on postoperative day 2.

Surgical Approach

Endovascular access was gained using a modified Seldinger technique by a single-wall puncture of the right common femoral artery with a 4-French micropuncture set. A 6 French sheath was then introduced into the right common femoral artery. An expanded EEA was performed. The head was fixed in three-point fixation. Neuronavigation was set up. Neuro-monitoring for the left cranial nerves III and VI was set up. A left middle turbinectomy was performed. Since the patient had a septal perforation, an inferior turbinate flap was raised. The sella, tuberculum, planum, left parasellar and paraclival carotids, and left optic canal were exposed using a 4 mm diamond drill. The left cavernous sinus was opened with sharp dissection, rostrally up to the proximal dural ring and distally toward the foramen lacerum. Venous bleeding was controlled by embolizing the cavernous sinus with a flowable gelatin matrix. This is a critical step to gain access to the cavernous carotid artery for proximal control. 

Aneurysm Exposure and Clipping

Next, the suprasellar dura mater was opened in the usual fashion. The left optic sheath was opened to free the left optic nerve and facilitate its mobilization. The distal ring and arachnoid adhesions were sharply dissected. A large aneurysm lateral to the optic nerve was identified (Figure 3). Further dissection was performed to fully expose the wide neck of the aneurysm by gently lifting the optic nerve (Figure 3). A temporary clip was applied for approximately two minutes to the paraclival cavernous carotid artery to soften the aneurysm (Figure 3). Under direct visualization, a single bayonet clip was applied under the optic nerve (Figure 4). Clip ends were carefully inspected to ensure that the entire neck was secured. The temporary clip was then removed. Intraoperative angiography was performed using a portable C-arm to confirm the absence of any residual neck and to ensure no compromise of the ophthalmic artery or parent vessel (Figure 4). 

**Figure 3:**
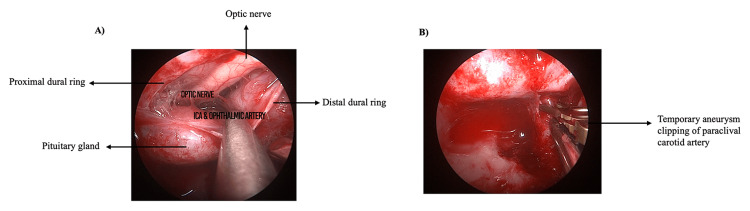
A) Endoscopic endonasal view of key structures identified prior to clipping of the ophthalmic artery aneurysm. B) Proximal control was obtained by placing a temporary aneurysm clip on the paraclival carotid artery.

**Figure 4:**
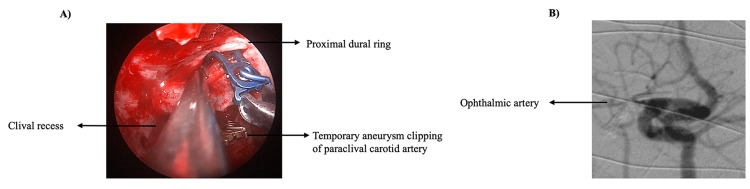
A) A single bayonet aneurysm clip was applied over the ophthalmic artery aneurysm. B) Lateral digital subtraction angiography of the right internal carotid artery injection following aneurysm clipping, showing patency of the ophthalmic artery.

Closure

The suprasellar space was packed with fat. Fat was placed around the clip in the sphenoid sinus. This was covered with fascia lata, and a vascularized inferior turbinate flap was placed over the fascia. No cerebrospinal fluid (CSF) diversion was used. Estimated blood loss was 50 cc, and the length of the case was approximately six hours. The patient had an excellent outcome without any neurological deficits and was discharged home on postoperative day 2. A non-contrast head CT scan obtained one week postoperatively showed no complications and resolution of pneumocephalus (Figure 5).

**Figure 5:**
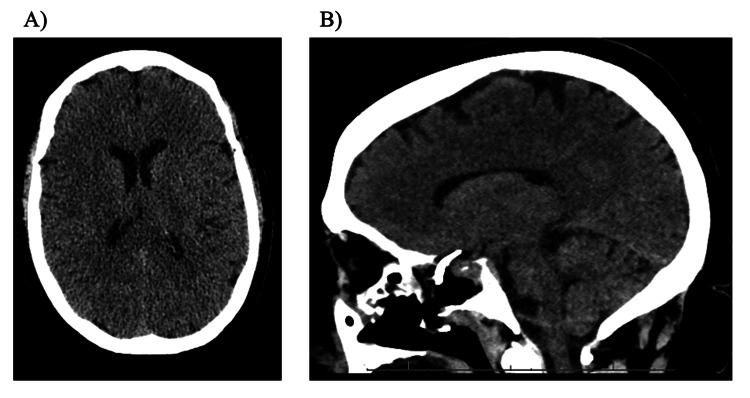
(A-B) One week post-operatively non-contrast head computed tomography (CT) scan showing lack of pneumocephalus.

## Discussion

CAs pose significant morbidity and mortality, especially in younger patients [3,18-20]. Endovascular surgery has revolutionized the treatment of CAs, but current treatment options are limited by relatively high recurrence rates and the need for dual antiplatelet therapy (DAT) postoperatively [21-24]. On the contrary, CA clipping has a much lower risk of recurrence [25-28] but requires a steep learning curve. This is especially true for more complex and rare CAs, such as posterior circulation and paraclinoid aneurysms [29-31]. 

Transcranial approaches for aneurysm clipping are well established. These approaches fulfill the basic requirements of aneurysm surgery, including wide exposure and proximal and distal vascular control. These traditional approaches have been further advanced with minimally invasive keyhole approaches for certain aneurysms, which have added the benefit of excellent cosmetic results but require advanced skills and familiarity with surgical anatomy [32,33]. Recently, EEA has been used to clip carefully selected CAs in certain locations in well-selected patients [6,7,10-12,14,15,17]. Limited case reports and case series of CA clipping in both the anterior and posterior circulation have demonstrated the feasibility and challenges of this complex and advanced approach.

In this case report, we describe our experience and technical nuances of clipping an OAA lateral to the optic nerve using an expanded EEA. Our patient was a 36-year-old female who presented with bilateral OAA aneurysms: a larger 7 × 7 mm aneurysm with a 4 mm neck and a daughter sac on the left, and a smaller 2 × 3 mm wide-neck aneurysm on the right. Aneurysm coiling would be challenging in this situation due to the wide neck of the aneurysm. A flow diverter could have been a reasonable option; however, concerns included her young age and bilateral aneurysms. If a flow diverter were placed for the larger left-sided aneurysm, she would require DAT in the short term and prolonged use of at least one antiplatelet agent. Furthermore, she would be at higher risk of aneurysm growth and potential rupture of the right-sided OAA, which might then require another flow diverter. Bilateral flow diverters in both carotid arteries would have placed this young patient at significant risk of long-term complications.

A traditional open craniotomy could have been an option here. Although most vascular surgeons are familiar with such approaches, the treatment of deeply located OAAs is not without its own challenges [34,35]. Even after an anterior clinoidectomy, proximal control can be challenging due to the close proximity of the distal dural ring. Identifying the origin of the ophthalmic artery is difficult. Further, there is significant brain retraction and manipulation involved. In addition, cosmetic outcomes may not be insignificant, as patients place a very high priority on them.

EEA presents its own benefits and challenges [36,37]. The most important benefit is that it provides direct access to the aneurysm and excellent proximal vascular control. Accessing OAAs through the sphenoid sinus provides a significantly shorter distance. In addition, EEA provides direct visualization of all critical structures, including the clinoidal segment of the carotid artery, distal dural ring, ophthalmic artery origin, and optic nerve. One critical challenge in this particular case was the superior location of the OAA. Previously published cases have used EEA to clip medially directed OAAs. A medially directed aneurysm provides relatively direct access to the aneurysm neck without manipulation of the optic nerve. An OAA lateral to the optic nerve means that the optic nerve blocks the view of the aneurysm neck. This requires optic nerve manipulation to visualize the full extent of a wide-necked aneurysm. During our initial exposure, we could only visualize the medial aspect of the neck of the aneurysm without elevating the optic nerve more superiorly. To minimize manipulation, we drilled the full length of the bony optic canal and opened the optic sheath to free the optic nerve. This allowed us to gently elevate the optic nerve and visualize the full extent of the wide neck of this aneurysm. Care must be taken not to apply a straight or curved clip in superiorly directed OAAs, as the clip may recoil and exert pressure on the optic nerve after application. We used a bayoneted clip, which provides better visualization of critical structures and does not recoil to put pressure on the optic nerve. Care must also be taken not to injure the third cranial nerve running lateral to the neck of the aneurysm. Therefore, electrophysiological monitoring of the third nerve is essential. Our patient did not experience any vision-related changes postoperatively. 

A critical benefit of this approach is that no brain retraction is required to access this aneurysm, which would otherwise be unavoidable if a transcranial approach had been adopted [38,39]. Open approaches often require the use of fixed retractors. The use of such retractors has well-known complications, such as postoperative cerebral edema and focal ischemic changes [39,40]. Our approach resulted in rapid recovery from surgery without any damage to critical brain structures.

EEA avoids another challenge of the open approach: visualization of the ophthalmic artery origin. EEA provides early identification of the ophthalmic artery origin, and it remains under direct visualization throughout the process of clip application. The same is true for the internal carotid artery. Not only does it provide an excellent view of the various segments of the carotid artery, but it also provides access to a long segment of the cavernous paraclival carotid artery for proximal control. We applied a temporary clip to this segment of the carotid artery prior to permanent aneurysm clipping to soften this large aneurysm, allowing good access to the neck and minimizing manipulation of the optic nerve. Care must be taken not to inadvertently clip the sixth cranial nerve, which courses posterior to the paraclival carotid artery on its way to the cavernous sinus. We monitored the sixth nerve in this case. 

CSF leak remains a challenge for expanded EEA intradural procedures. Also, the clip protrudes into the sphenoid sinus. The typical repair process has been to apply fascia lata first, followed by a vascularized flap [41]. There is also a risk of clip erosion through the fascia and the flap. To minimize the risk of CSF leak and clip erosion, we placed fat in the suprasellar space, taking care not to overpack, which can cause compression of the optic apparatus. Fat is also placed around the clip in the sphenoid sinus. This is then covered with fascia and a vascularized flap. No CSF diversion was used. No postoperative CSF-related complications were noted.

Familiarity with the expanded EEA and anatomy is critical. Operating in a restrictive space can be challenging. Handling vascular pathologies through EEAs is the peak of the experience curve [7]. A multidisciplinary skull base operating room team is critical to ensuring good outcomes. Specialized instruments are needed. We used a clip applier that holds the clip inside out and provides better visualization than standard appliers. Patients should be counselled regarding their options without bias.

## Conclusions

EEA provides an excellent and direct corridor for OAA clipping that is lateral to the optic nerve without any cosmetic defects. Extensive experience with EEAs and familiarity with intradural and extradural neurovascular structures is critical to ensuring good surgical outcomes.
